# Development of the Clinical Interpersonal Reactivity Index to evaluate nurses’ empathy

**DOI:** 10.1111/nhs.12875

**Published:** 2021-09-15

**Authors:** Yoshimi Aoki, Harumi Katayama

**Affiliations:** ^1^ Department of Fundamental Nursing Hamamatsu University School of Medicine Shizuoka Japan

**Keywords:** communication, cross‐sectional study, empathy, Japan, nurses

## Abstract

We determined the validity and reliability of the Clinical Interpersonal Reactivity Index in a sample of Japanese nurses. Participants were registered nurses at national university hospitals and nursing researchers in Japan. A postal questionnaire was conducted. Construct validity was analyzed by exploratory and confirmatory factor analysis, and convergent validity demonstrated using the Interpersonal Reactivity Index. The Clinical Interpersonal Reactivity Index has an 18‐item, two‐factor structure with Cronbach's alpha values of 0.87 and 0.73. Confirmatory factor analysis showed a goodness‐of‐fit index of 0.917, an adjusted goodness‐of‐fit of 0.894, a root mean square error of approximation of 0.60, and a comparative fit index of 0.911. Correlation analysis between the Clinical Interpersonal Reactivity Index and Interpersonal Reactivity Index indicated the factors were significantly correlated for empathic concern and perspective taking (*r* = 0.439–0.401). Test–retest assessment showed reliability coefficients for the first factor as *r* = 0.859 and the second factor as *r* = 0.709. The Clinical Interpersonal Reactivity Index demonstrated validity and reliability for Japanese nurses. The two factors evaluated perspective taking and unconditional positive regard.

**Key points**

- The Clinical Interpersonal Reactivity Index was developed to evaluate nurses’ empathy, an essential part of bedside communication.
- This was a cross‐sectional, psychometric, instrumental study.
- The validity and reliability of the Clinical Interpersonal Reactivity Index were confirmed.

## 1 INTRODUCTION AND BACKGROUND

There is an ongoing debate about empathy. Definitions of empathy may refer to the underlying abilities or actions that enable the experience of empathy (van Dijke et al., 2020). Empathy offers a direct and immediate form of other‐understanding, and does not require us to reproduce or share the other's experience (Fernandez & Zahavi, 2020). Empathy is also a concept closely related to compassion (Hojat, 2016; Sinclair et al., 2016) and active listening (Asai et al., 2020; Rogers, 1957). Compassion is included in the emotional component of empathy (Hojat, 2016). It is characterized by feelings of warmth, concern, and care for the other, as well as a strong motivation to improve the other's well‐being (Singer & Klimecki, 2014). The indispensable method for experiencing empathy is active listening (Rogers, 1957, 1975). Rogers (1975) states that empathic understanding and active listening (absolute listening) are similar and complex concepts that occur at the same time in effective counseling. It is important for nurses to show empathy in communicating with patients when caring for them (Bullington et al., 2019; Oh, 2019).

Davis (1996), a social psychologist, has reported on multifaceted empathy with emotional and cognitive components. In many situations, the word empathy is used in the context of the emotional component of sharing the same feelings as the other person. However, the empathy required of health professionals also involves the cognitive component of thinking by putting oneself in the patient's shoes (Hojat, 2016; McKinnon, 2018). Rogers (1957), who showed the importance of empathy in patient‐centered therapy, stated that empathic understanding is to sense the client's private world as if it were your own but without ever losing the “as if” quality; this is empathy, and this seems essential to therapy. The empathic understanding shown by Rogers (1957) is considered the cognitive component of empathy shown by Davis (1996). The emotional and cognitive components are often confused (Gerace, 2020). Therefore, the social psychologist Davis (1983) showed the characteristics of empathy as a multidimensional concept. He said that the interpersonal empathy necessary for health care is “perspective taking” among the cognitive components of empathy (Davis, 1996). Davis (1996) also said that the most advanced process is what has been termed perspective taking: the attempts by one individual to understand another by imagining the other's perspective. It is typically an effortful process, involving both the suppression of one's own egocentric perspective on events and the active entertaining of someone else's perspective (Davis, 1996). Fernandez and Zahavi (2020) showed nurses’ basic empathy, which amounts to something akin to perspective taking, in which nurses attempt to put patients in the place of the other, yet abstain from fully identifying with them.

The concept of empathy among nurses is vague and confusing, and many nurses confuse cognitive empathy with emotional empathy (Aoki & Katayama, 2019; Fernandez & Zahavi, 2020). It has been shown that emergency department nurses have difficulty showing empathy towards patients who self‐harm (Aoki & Katayama, 2017; Saunders et al., 2012). It has also been shown that clinical nurses have difficulty empathizing with people with dementia (Digby et al., 2017; Teófilo et al., 2019). Educational programs that include knowledge sharing and simulations have been implemented to help nurses effectively acquire and use empathy (Bas‐Sarmiento et al., 2019; Yang et al., 2020; Levett‐Jones et al., 2019). To develop those programs, we need an index for evaluating the learner's empathy.

Many measures of empathy have been developed so far (Everson et al., 2018; Levett‐Jones et al., 2019; Yu & Kirk, 2009). Among them, the most frequently used are the Hogan Empathy Scale (HES; Hogan, 1969), the Interpersonal Reactivity Index (IRI; Davis, 1983), the Empathy Construct Rating Scale (ECRS; La Monica, 1981), the Reynolds Empathy Scale (RES; Reynolds, 2000), the Barrett‐Lennard Relationship Inventory (BLRI; Barrett‐Lennard, 2015), and the Jefferson Scale of Empathy (JSE; Hojat, 2016). The HES and IRI were developed to evaluate empathy in general interpersonal relationships (Davis, 1983; Hogan, 1969). The full version of the BLRI consists of 64 items (short version: 40 items) that evaluate empathic understanding as one of the necessary conditions for the therapist (Barrett‐Lennard, 2015). The BLRI has been translated into Japanese and that version has 20 items (Ikemi et al., 2001). The BLRI is effective in assessing long‐time interactions, but has difficulty in assessing short‐term, repetitive interactions between nurses and patients. The ECRS and RES were developed to evaluate empathy in clinical nurses (La Monica, 1981; Reynolds, 2000). However, these scales have numerous items and explanatory texts, and it is difficult to evaluate them in a short time. The ECRS has 84 items and the RES has 12 items (totalling approximately 2500 words, including explanatory documents). The JSE is an internationally usable measure (Maximiano‐Barreto et al., 2020) which includes items that can evaluate perspective taking in health professionals (Hojat, 2016; Kataoka et al., 2018), but it does not contain items that evaluate specific helping actions.

Nonverbal communication required for empathy has been reported to be influenced by culture (Lorié et al., 2017). There are also reports of a strong correlation between cultural considerations and empathy (Sharifi et al., 2019; Zarei et al., 2019). The previously developed measures may not be suitable for evaluating empathy in the Japanese.

The purpose of this study was to develop a scale that easily evaluates Japanese nurses’ perspective taking, which reflects their empathic abilities and related helping actions. Indicators that assess the ability of cognitive empathy can help in the development of practical and effective education programs.

## 2 METHODS

### 2.1 Definition of terms

In this study, we defined empathy as an ability or action which enables the experience of other‐understanding, while not requiring the replication or sharing of the other's experience. The Clinical Interpersonal Reactivity Index (CIRI) was developed to evaluate empathy as a reaction in interpersonal relationships at the bedside.

### 2.2 Study design and sample

This cross‐sectional psychometric instrumental study determined the reliability and validity of the CIRI in a sample of Japanese registered nurses. The study was carried out from February to October of 2019. The participants were registered nurses belonging to national university hospitals and nursing researchers in Japan. We limited selection to persons with more than 5 years of nursing experience, because in order to develop an index that evaluates nurses' empathy, we wanted the participants to be nurses with many years of experience who could be considered to have established an identity as a nurse, and were accustomed to communicating with patients.

### 2.3 Instrument

The draft Clinical Interpersonal Reactivity Index (CIRI‐D) has 27 items that evaluate empathy in nurses (Aoki & Katayama, 2019). The CIRI‐D items were developed to evaluate empathy in the following way (Aoki & Katayama, 2019). The authors conducted semi‐structured interviews with five nurses who were able to talk about empathy and who were recommended by a facility administrator. All participants were female and aged between 34 and 64 years (average age, 47.4 years). The author explained to the participants that empathy is an experience of understanding the behavior of others, not sharing that experience, before the interview. The data were analyzed qualitatively and subjected to descriptive analysis. The rigor of the items was verified by comparing them with two existing theories, a nursing theory by Travelbee (1971) and a psychological theory by Rogers (1957). Based on the clinical scenes described by the participants, items about empathy were extracted, and it was confirmed that the items were consistent with the existing theory. As a result of this study, 27 items were thus developed, consisting of four phases or conditions. Travelbee (1971) stated that a human‐to‐human relationship is established after a nurse and the recipient in her care have progressed through four preceding interlocking phases. These phases are the original encounter, emerging identities, empathy, and sympathy (Travelbee, 1971). Travelbee (1971, p. 150) said that “all of these phases culminate in rapport and the establishment of the human‐to‐human relationship.” Rogers (1957) listed six conditions as a process necessary for effective psychotherapy. These conditions are also common to the process of establishing interpersonal relationships between patients and nurses that include empathy. The current analysis used four of these conditions, which were considered compatible with items extracted from the interview contents. The four conditions were as follows: “Two persons are in psychological contact”; “The second person, whom we shall term the therapist, is congruent or integrated in the relationship”; “The therapist experiences unconditional positive regard for the client”; and “The therapist experiences an empathic understanding of the client's internal frame of reference and endeavors to communicate this experience to the client” (Rogers, 1957, p. 96).

The dependability of all items was confirmed since they conformed to existing theories, and their credibility was confirmed by discussion between eight nursing researchers, including the authors (January 25, 2018). We presented the items so that the respondent selects the answer that best describes his or her personal situation from a Likert scale with four choices (1 = extremely unlikely; 2 = moderately unlikely; 3 = moderately likely; 4 = extremely likely). As much as possible, the number of points was set as low as 4 points so that the evaluation would not take much time. Since there are many items asking about the interest of the patient, and a neutral answer is regarded as “not interested” and cannot be evaluated, we used an even‐numbered Likert scale.

The content validity of the scale had already been verified (Aoki & Katayama, 2019). The pretest was carried out from September to October of 2018. The participants responded to the pretest online. The participants (*n* = 58) were nurses working in psychiatric hospitals, nurses providing terminal care, and nurses working in emergency departments in Japan, since these areas are considered to require high empathy. The participants had more than 5 years of clinical experience. Based on the results, four items were revised because inter‐item correlation was not achieved. The authors discussed the content validity of the four revised items. Then, the face validity was discussed at two meetings of experts.

In this study, the IRI was used to examine the construct validity of the CIRI. The Japanese version of the IRI has 26 items that evaluate empathy in nurses (Himichi et al., 2017). The IRI is a scale developed to evaluate empathy in general interpersonal relationships in multiple dimensions (Davis, 1980, 1996). The respondent selects the answer that best describes his or her personal situation from a 4‐point Likert scale (from 1 = extremely unlikely, to 4 = extremely likely). We used the Japanese version and the original version of the IRI with permission from the authors.

There are four subscales in the IRI. The empathic concern scale assesses the tendency to experience feelings of sympathy and compassion for others in unfortunate situations (Davis, 1996). The perspective taking scale measures the reported tendency to spontaneously adopt the psychological point of view of others in everyday life (Davis, 1996). The personal distress scale gauges the tendency to experience distress and discomfort in response to extreme distress in others (Davis, 1996). The fantasy scale measures the tendency to imaginatively transpose oneself into fictional situations (Davis, 1996).

### 2.4 Procedure for data collection

The facilities for data collection were 44 national university hospitals in Japan (excluding Hospital A, discussed below). The request to participate in this study was made by telephone and cover letter to the facilities in June 2019. The questionnaires were mailed to facilities that agreed to participate, and ward managers were requested to distribute the questionnaire to nurses who had more than 5 years of experience. The questionnaire was distributed and collected by postal mail individually. The collection period was from July to October.

The test–retest reliability assessment was carried out from February to April 2019. The participants were 20 nurses working in the psychiatric ward of a national university hospital (Hospital A), and 11 nursing researchers participating as experts. The nursing researchers had more than 5 years of clinical experience and were involved in clinical practice and instructing students in clinical training. The retest was conducted 2 weeks after the first test.

### 2.5 Statistical analysis

The analysis was carried out according to *Health Measurement Scales: A Practical Guide to Their Development and Use* (5th ed.; Streiner et al., 2015). SPSS for Windows ver. 26, and Amos for Windows ver. 26, were used for the analysis.

First, descriptive statistics on the background of the participants were collected. Second, the mean value was substituted for any missing value, and reverse items were processed. After that, the ceiling and floor effects, the item‐total correlation, Cronbach's α coefficient when the item was eliminated, and the number of missing items were confirmed for each item of the CIRI‐D. The item‐total correlation was used as a criterion for excluding items with an α value of ≤0.3 or ≥0.7. Third, exploratory factor analysis and confirmatory factor analysis were carried out. The exploratory factor analysis was used as a criterion for excluding items with a factor loading of ≤0.400. The validity of the CIRI composed of CIRI‐D items was confirmed by confirmatory factor analysis. Fourth, convergent validity of the CIRI factors was established through correlation with the IRI subscale. Fifth, the test–retest reliability assessment was made by calculating the correlation coefficient for the results of the first and second tests.

### 2.6 Ethical considerations

The study was approved by the clinical research ethics committee of the Hamamatsu University School of Medicine (18–267; January 30, 2020–August 31, 2020). No personal data were collected that would allow the identification of the nurses, and participation was completely voluntary and anonymous. The study complied with current legislation in Japan on ethical guidelines for medical research on humans, and the ethical principles of the Declaration of Helsinki were respected at all times.

## 3 RESULTS

### 3.1 Participants’ background

The questionnaires were distributed to 819 nurses in 23 facilities. As a result, 402 nurses responded to the questionnaire (return rate: 49.1%). Of these, two participants with many missing values and one participant with less than 2 years of clinical experience were excluded, leaving 399 valid responses (valid response rate: 48.7%). Six participants who responded that they had 4 years of clinical experience were judged to have approximately 5 years of experience and were not excluded.

The participants were 369 women (92.5%), and had a mean age of 38.3 years (SD = 9.1). Of these, 169 participants (42.4%) responded that their highest level of education was a four‐year college (Table 1).

**TABLE 1:** Background of the participants (*n* = 399)

Item	Mean (SD)	Number (%)
Gender		
Female		369 (92.5)
Male		29 (7.3)
No answer		1 (0.3)
Age	38.3 (9.1)	
Clinical experience years	15.7 (9.0)	
Final education		
College		169 (42.4)
Diploma programs		146 (36.6)
Junior college		43 (10.8)
Graduate school		32 (8.0)
Other		7 (1.8)
No answer		2 (0.5)

*Note*: All participants had qualifications as registered nurses.

### 3.2 Item selection

As a result of the analysis, nine CIRI‐D items were excluded (Table 2). Item 9 was excluded because a ceiling effect was observed. Items 4, 7, 18, 21, and 22 were excluded because the item‐total correlation was 0.3 or less. In addition, Items 3 and 14 were excluded because Cronbach's α coefficient was 0.86 or more when these items were eliminated. Exploratory factor analysis was carried out using the maximum likelihood method and promax rotation. The scree plot confirmed the number of factors and components. As a result, Item 1 was excluded because the factor loading did not clear the cutoff of 0.400.

**TABLE 2:** Descriptive results of the draft Clinical Interpersonal Reactivity Index (*n* = 399)

		Mean	SD	Item‐total correlation	Cronbach's coefficient α when the item was eliminated	Number missing
1	I wait for patients to talk naturally, and I often visit them in their room.	2.63	0.62	0.36	0.85	0
2	To make a relationship of trust, I am actively talking to patients.	3.20	0.58	0.56	0.85	0
3	I can talk to my boss about patient relationships.	3.04	0.72	0.30	0.86	0
4	^a^I think that I can understand patients by experiencing the same diseases and sufferings as patients.	2.48	0.75	‐0.06	0.87	5
5	^a^I cannot understand patients who make unreasonable demands or exhibit troublesome behavior.	2.87	0.66	0.37	0.85	0
6	I try to accept the experiences that the patient tells me about.	2.85	0.64	0.41	0.85	2
7	I think it is necessary to let another nurse handle my patient when I feel negatively towards the patient and it is difficult for me to talk with them.	3.27	0.59	0.15	0.86	0
8	I reflect on my attitude towards patients and try to improve it if necessary.	3.32	0.54	0.43	0.85	0
9	I think that when a patient consults me, there are cases in which he/she purely wants only to talk with me.	3.56	0.52	0.48	0.85	0
10	I sometimes explain to patients the current busy situation frankly and gently, and adjust the time to hear their story.	3.06	0.58	0.36	0.85	1
11	I think that there are reasons for violence, abuse, and refusals from patients.	3.15	0.55	0.51	0.85	0
12	Even when a patient refuses my involvement, I want to understand their feelings.	3.04	0.64	0.48	0.85	0
13	When I provide guidance to a patient, I try to show them that I understand their feelings.	3.12	0.52	0.50	0.85	1
14	^a^I often doubt and cannot understand patients' stories.	2.74	0.61	0.21	0.86	1
15	I try to create an environment where patients can relax and talk.	3.20	0.52	0.56	0.85	0
16	I talk to patients in a kind tone and listen to their stories.	3.30	0.53	0.49	0.85	1
17	When interacting with patients, I try to imagine their feelings and tell them what I think.	3.05	0.55	0.52	0.85	0
18	^a^I think it is important to give advice to patients.	2.57	0.58	‐0.06	0.86	1
19	I want to actively learn about my patients.	3.06	0.62	0.59	0.85	1
20	Although I may not know the exact amount of suffering that the patient is experiencing, I tell them that I understand their suffering.	3.24	0.53	0.56	0.85	1
21	^a^I feel irritated when patients use the nurse call repeatedly while I am busy.	2.28	0.71	0.26	0.86	2
22	When I cannot understand a patient's feelings, I consider that the reason for this may be their disease and symptoms.	2.90	0.64	0.22	0.86	0
23	Although I cannot put myself in a patient's shoes, I try to imagine and understand their feelings as best I can.	3.29	0.54	0.63	0.84	0
24	To understand the patient, I think it is important to grasp their experiences, behavior, expressions, and life rhythm as a whole.	3.43	0.52	0.61	0.85	0
25	The patients and I search for solutions to their problems together.	3.12	0.49	0.58	0.85	0
26	I want all patients to recover, even those who insulted or were violent towards me.	3.01	0.64	0.47	0.85	1
27	I adjust my schedule and prepare myself emotionally so that I can listen to a patient's story.	3.15	0.56	0.60	0.85	0

*Note*: Items are in Japanese.

^a^ Reverse‐scored item.

**FIGURE 1:**
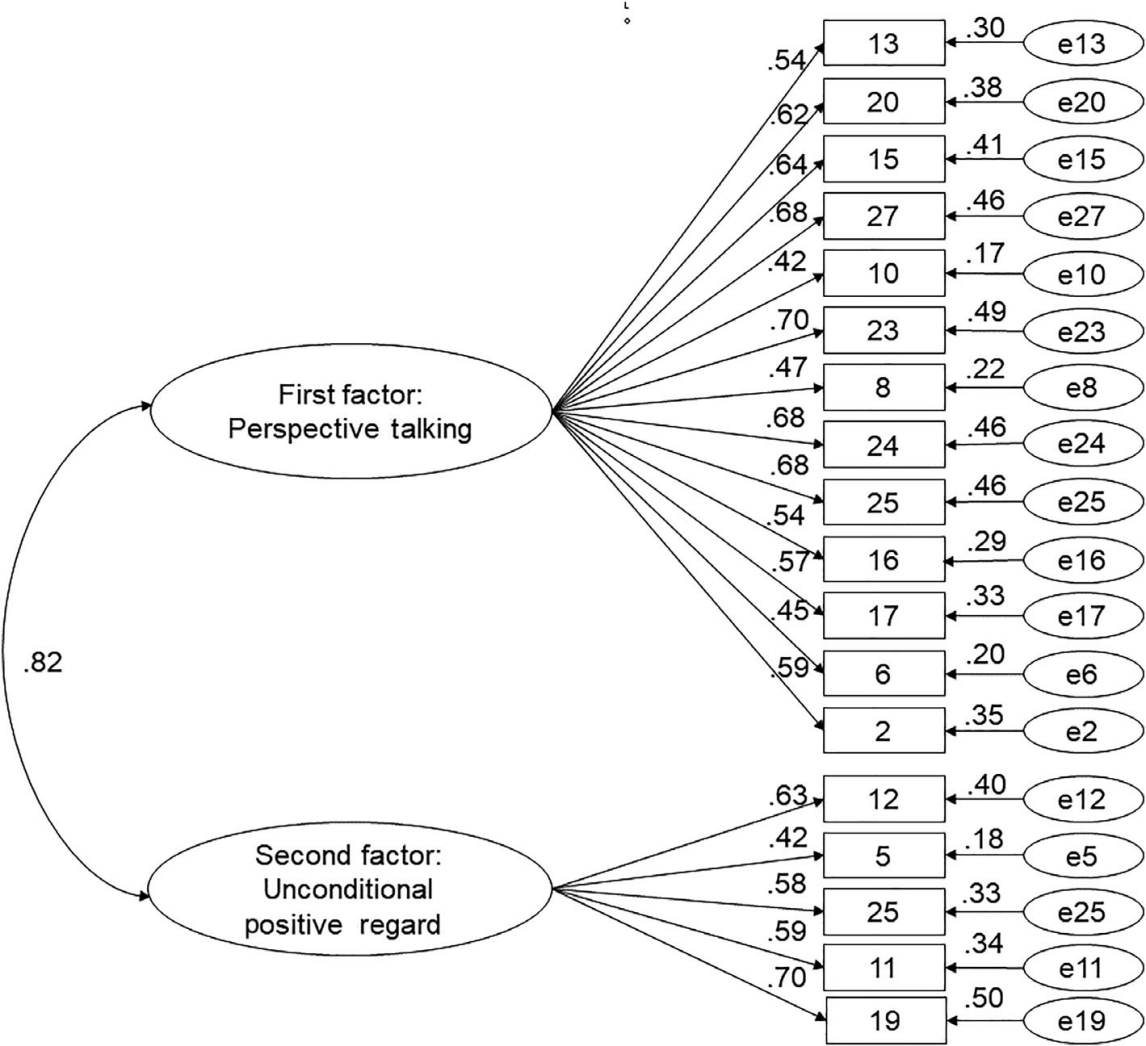
Confirmatory factor analysis of the Clinical Interpersonal Reactivity Index (*n* = 399)

The CIRI has 18 items with a two‐factor structure. Cronbach's α coefficient was 0.89 for the overall scale, 0.87 for the first factor, and 0.73 for the second factor (Table 3). As a result of confirmatory factor analysis using 399 participants, the goodness‐of‐fit index (GFI) was 0.917, adjusted goodness‐of‐fit index (AGFI) was 0.894, comparative fit index (CFI) was 0.911, and root mean square error of approximation (RMSEA) was 0.060 (Figure 1).

**TABLE 3:** Exploratory factor analysis of the Clinical Interpersonal Reactivity Index (*n* = 399)

Item		Factors	
		l	2
First factor: Perspective taking			
13	When I provide guidance to a patient, I try to show them that I understand their feelings.	**0.677**	−0.148
20	Although I may not know the exact amount of suffering that the patient is experiencing, I tell them that I understand their suffering.	**0.667**	−0.049
15	I try to create an environment where patients can relax and talk.	**0.647**	0.001
27	I adjust my schedule and prepare myself emotionally so that I can listen to a patient's story.	**0.581**	0.122
10	I sometimes explain to patients the current busy situation frankly and gently, and adjust the time to hear their story.	**0.580**	−0.193
23	Although I cannot put myself in a patient's shoes, I try to imagine and understand their feelings as best I can.	**0.577**	0.150
8	I reflect on my attitude towards patients and try to improve it if necessary.	**0.568**	−0.106
24	To understand the patient, I think it is important to grasp their experiences, behavior, expressions, and life rhythm as a whole.	**0.547**	0.160
25	The patients and I search for solutions to their problems together.	**0.535**	0.173
16	I talk to patients in a kind tone and listen to their stories.	**0.503**	0.051
17	When interacting with patients, I try to imagine their feelings and tell them what I think.	**0.494**	0.097
6	I try to accept the experiences that the patient tells me about.	**0.475**	−0.030
2	To make a relationship of trust, I am actively talking to patients.	**0.428**	0.206
Second factor: Unconditional positive regard			
12	Even when a patient refuses my involvement, I want to understand their feelings.	−0.064	**0.690**
5	^a^I cannot understand patients who make unreasonable demands or exhibit troublesome behavior.	−0.227	**0.657**
26	I want all patients to recover, even those who insulted or were violent towards me.	−0.047	**0.644**
11	I think that there are reasons for violence, abuse, and refusals from patients.	0.063	**0.542**
19	I want to actively learn about my patients.	0.315	**0.419**
	Cronbach's coefficient α	0.87	0.73
	Inter‐factor correlation		0.729

*Note*: Maximum likelihood method was used with oblique rotation (promax). Items are in Japanese. Factor loadings greater than .400 are shown in bold.

^a^ Reverse‐scored item.

### 3.3 Construct validity

Correlation analysis showed a significant correlation between the CIRI and subscales of the IRI. The first factor of the CIRI was significantly correlated with the empathic concern scale of the IRI (*r* = 0.439) and perspective taking of the IRI (*r* = 0.401; *p* ≤ 0.01). The second factor of the CIRI was significantly correlated with the empathic concern scale of the IRI (*r* = 0.418) and the perspective taking scale of the IRI (*r* = 0.375; *p* ≤ 0.01; Table 4).

**TABLE 4:** Pearson's correlation coefficient between the Interpersonal Reactivity Index and the Clinical Interpersonal Reactivity Index (*n* = 399)

	Interpersonal Reactivity Index (Japanese version)			
	Personal distress	Empathic concern	Perspective taking	Fantasy
Clinical Interpersonal Reactivity Index				
First factor (perspective taking)	−0.130**	0.439**	0.401**	0.108*
Second factor (unconditional positive regard)	−0.192**	0.418**	0.375**	0.087

* *p* ≤ 0.05.

** *p* ≤ 0.01.

### 3.4 Test–retest reliability

The participants in the test–retest assessment were 20 nurses and 11 nursing researchers. There were 30 valid responses (28 women and two men; Table 5). As a result of correlation analysis of the first and second tests, the overall scale had a reliability coefficient of *r* = 0.843 (*p* ≤ 0.01), with the first factor as *r* = 0.859 (*p* ≤ 0.01), and the second factor as *r* = 0.709 (*p* ≤ 0.01).

**TABLE 5:** Backgrounds of test–retest participants (*n* = 30)

Item	Mean (SD)	Number
Gender		
Female		28
Male		2
Age	37.2 (10.77)	
Clinical experience years	13.0 (10.89)	
Final education		
Diploma programs		9
Junior college		2
College		14
Graduate school		5

*Note*: All participants had qualifications as registered nurses.

## 4 DISCUSSION

### 4.1 Suitability

As a result of distributing questionnaires to 819 nurses working at national university hospitals in Japan, 399 valid responses were returned. The target population of this study was Japanese nurses. There are approximately 1 660 000 nurses in Japan (Japanese Nursing Association, 2018). The recovery rate for this study was 49.1%, the confidence interval was 95%, and the margin of error was 5%. These results are considered to reflect the general population of nurses in Japan. In addition, the participants had more than 5 years of experience as nurses and are accustomed to empathic communication with patients. The most common age group of Japanese nurses is between 40 and 44 years (15.0%), followed by 35 to 39 years (14.3%; Japanese Nursing Association, 2016). The mean age of the nurses in this study was 38.3 years, which reflects the mean age of Japanese nurses overall. For the above reasons, the data in this study are considered appropriate for examining the reliability and validity of the CIRI for evaluating the empathy of Japanese nurses.

### 4.2 Scale reliability

The CIRI showed an overall Cronbach's α coefficient of 0.89, indicating good reliability. The Cronbach's α coefficient for each individual factor was 0.70 or higher; therefore, the CIRI was again found to be reliable.

For test–retest reliability, the correlation between the first and second tests should be 0.75 or higher. The first factor met this condition, but the second factor did not. The reason may be that the second factor is related to an individual nurse's own psychological state, which may have changed over the 2‐week interval. From the above, it may be considered that the reliability of both the first factor and the second factor was also confirmed.

### 4.3 Scale validity

We examined content validity during scale creation. The face validity, readability, and ambiguity of each question were discussed several times while developing the item before pretest. Eleven nursing researchers provided objective evidence of each item's suitability.

We considered the factorial validity of the scale using exploratory factor analysis. The factor structure of the CIRI had 18 items in two factors. Through goodness of fit analysis, we concluded that the model had good explanatory power, with index values of GFI = 0.917 and AGFI = 0.894. Both of these values easily meet the standard criteria for goodness of fit (GFI > 0.9, GFI > AGFI). The RMSEA (0.060) and CFI (0.911) values were also well beyond the range of cutoff values for poor fit (RMSEA > 0.1, CFI < 0.9). Therefore, our model had no problems with factor suitability. These procedures ensured the validity of the CIRI.

The subscales of the CIRI were significantly correlated with the empathic concern and perspective taking scales of the IRI. The second factor had a weak correlation with the empathic concern scale (*r* < 0.400). The empathic concern and perspective taking scales have been shown to be associated with helping behavior (Davis, 1996). Social psychologist Batson (2011) characterized empathic concern (empathy) as other‐oriented emotion elicited by and congruent with the perceived welfare of someone in need. He cited perspective taking as one of the cognitive and perceptual states of empathic concern, and further stated that the condition is likely to be a precursor or facilitator of empathic concern (Batson, 2011). Thus, the subscales of the CIRI were considered to be related to the empathic concern and perspective taking scales because they evaluated the empathy of the helping behavior of nurses.

The subscales of the CIRI did not correlate with the personal distress scale of the IRI. Davis (1996) has shown that empathic distress is not related to helping behavior. Batson (2011) also showed that empathic distress is not a step towards empathic concern. Therefore, the CIRI was not related to the personal distress scale.

The subscales of the CIRI were not correlated with the fantasy scale of the IRI. The fantasy scale, relative to the other three IRI scales, is difficult to fit into the constructs of empathy. Thinking by substituting oneself for a character in a fictional story, such as projection, may facilitate other‐oriented empathic concern (Batson, 2011). However, projection risks making a completely inaccurate interpretation of the other's state (Batson, 2011), and this risk has been shown to occur especially if we do not have a precise understanding of relevant self–other differences (Batson, 2011). Nurses are conscious of distinguishing themselves from others when empathizing with patients (Fernandez & Zahavi, 2020; Reynolds, 2000; Travelbee, 1971). Therefore, the fantasy scale was not related to the subscales of the CIRI.

Based on the contents of the items, the first factor evaluates perspective taking and the second factor evaluates unconditional positive regard. The first factor (perspective taking) includes 13 items that evaluate nurses’ empathy from the perspective of the nurse putting her/himself in the patient's shoes. These items can evaluate the nurse's perspective taking as a specific helping action. Perspective taking is a central concept of empathy in helping action, so it is considered appropriate to have more items than the second factor.

The second factor (unconditional positive regard) includes five items that evaluate nurses’ empathy towards patients and their desire to understand the patient's perspective. One among the five items is reverse‐scored. Rogers (1957) states that unconditional positive regard as a pre‐stage of empathy is a necessary condition of effective psychotherapy. Also, it is difficult to understand all patients without unconditional positive regard (Reynolds, 2000; Travelbee, 1971). The unconditional positive regard subscale includes items indicating nurses’ thoughts about the reasons for patient violence or refusal. The cognitive component of empathy is needed to focus on such patients. The unconditional positive regard scale was extracted as the cognitive component of empathy in helping actions.

### 4.4 Limitations

The CIRI has been translated into English; however, its reliability and validity have not yet been confirmed in English‐speaking countries. Furthermore, the CIRI was developed through the exclusive involvement of Japanese nurses. The CIRI has the potential to recognize the empathy of nurses around the globe and may contribute to the development of global empathic ability. Thus, it is necessary to investigate whether this scale can be used in other countries. We also only surveyed nurses. Future studies should investigate the applicability of the CIRI among nursing students. Moreover, the two factors of the CIRI did not have the same number of items, with the first factor (perspective taking) comprising 13 items and second factor (unconditional positive regard) comprising five items. In the future, it will be necessary to consider the number of items and aim for a well‐balanced structure.

## 5 CONCLUSIONS

The CIRI, a scale to measure and evaluate the empathy of Japanese nurses, was developed. The two‐factor, 18‐item CIRI developed in this study was confirmed as having sufficient reliability and validity across a variety of metrics. The CIRI can be used by nurses as a self‐assessment tool to evaluate their empathic perspectives and actions, and by nursing educators to evaluate the effectiveness of empathy education programs.

## CONFLICT OF INTERESTS

None.

## AUTHOR CONTRIBUTIONS

Study design: Yoshimi Aoki and Harumi Katayama.

Data collection: Yoshimi Aoki and Harumi Katayama.

Data analysis: Yoshimi Aoki and Harumi Katayama.

Manuscript writing: Yoshimi Aoki and Harumi Katayama

## Data Availability

Data available on request due to privacy/ethical restrictions
